# The genetic background of hydrocephalus in a population-based cohort: implication of ciliary involvement

**DOI:** 10.1093/braincomms/fcad004

**Published:** 2023-01-10

**Authors:** Tina N Munch, Paula L Hedley, Christian M Hagen, Marie Bækvad-Hansen, Frank Geller, Jonas Bybjerg-Grauholm, Merete Nordentoft, Anders D Børglum, Thomas M Werge, Mads Melbye, David M Hougaard, Lars A Larsen, Søren T Christensen, Michael Christiansen

**Affiliations:** Department of Epidemiology Research, Statens Serum Institut, DK-2300 Copenhagen, Denmark; Department of Neurosurgery, Copenhagen University Hospital, DK-2100 Copenhagen, Denmark; Department of Clinical Medicine, University of Copenhagen, DK-2100 Copenhagen, Denmark; Department for Congenital Disorders, Statens Serum Institut, DK-2300 Copenhagen, Denmark; The Lundbeck Foundation Initiative for Integrative Psychiatric Research, iPSYCH, DK-8000 Aarhus, Denmark; Brazen Bio, Los Angeles, 90502 CA, USA; Department for Congenital Disorders, Statens Serum Institut, DK-2300 Copenhagen, Denmark; The Lundbeck Foundation Initiative for Integrative Psychiatric Research, iPSYCH, DK-8000 Aarhus, Denmark; Department for Congenital Disorders, Statens Serum Institut, DK-2300 Copenhagen, Denmark; The Lundbeck Foundation Initiative for Integrative Psychiatric Research, iPSYCH, DK-8000 Aarhus, Denmark; Department of Epidemiology Research, Statens Serum Institut, DK-2300 Copenhagen, Denmark; Department for Congenital Disorders, Statens Serum Institut, DK-2300 Copenhagen, Denmark; The Lundbeck Foundation Initiative for Integrative Psychiatric Research, iPSYCH, DK-8000 Aarhus, Denmark; Department of Clinical Medicine, University of Copenhagen, DK-2100 Copenhagen, Denmark; The Lundbeck Foundation Initiative for Integrative Psychiatric Research, iPSYCH, DK-8000 Aarhus, Denmark; Mental Health Centre, Capital Region of Denmark, 2900 Hellerup, Denmark; The Lundbeck Foundation Initiative for Integrative Psychiatric Research, iPSYCH, DK-8000 Aarhus, Denmark; Center for Genomics and Personalized Medicine, Aarhus University, DK-8000 Aarhus, Denmark; Department of Biomedicine, Aarhus University, DK-8000 Aarhus, Denmark; The Lundbeck Foundation Initiative for Integrative Psychiatric Research, iPSYCH, DK-8000 Aarhus, Denmark; Mental Health Centre, Capital Region of Denmark, 2900 Hellerup, Denmark; Department of Clinical Medicine, University of Copenhagen, DK-2100 Copenhagen, Denmark; Department of Genetics, Stanford University School of Medicine, Stanford, CA 94305, USA; Centre for Fertility and Health, Norwegian Institute of Public Health, Oslo 0473, Norway; K.G. Jebsen Center for Genetic Epidemiology, Norwegian University of Science and Technology, Trondheim 7491, Norway; Department for Congenital Disorders, Statens Serum Institut, DK-2300 Copenhagen, Denmark; The Lundbeck Foundation Initiative for Integrative Psychiatric Research, iPSYCH, DK-8000 Aarhus, Denmark; Department of Cellular and Molecular Medicine, University of Copenhagen, DK-2100 Copenhagen, Denmark; Department of Biology, University of Copenhagen, DK-2100 Copenhagen, Denmark; Department for Congenital Disorders, Statens Serum Institut, DK-2300 Copenhagen, Denmark; The Lundbeck Foundation Initiative for Integrative Psychiatric Research, iPSYCH, DK-8000 Aarhus, Denmark; Department of Biomedical Science, University of Copenhagen, DK-2100 Copenhagen, Denmark

**Keywords:** hydrocephalus, ciliopathies, autism spectrum disorder, genetic, neurodevelopment

## Abstract

Hydrocephalus is one of the most common congenital disorders of the central nervous system and often displays psychiatric co-morbidities, in particular autism spectrum disorder. The disease mechanisms behind hydrocephalus are complex and not well understood, but some association with dysfunctional cilia in the brain ventricles and subarachnoid space has been indicated. A better understanding of the genetic aetiology of hydrocephalus, including the role of ciliopathies, may bring insights into a potentially shared genetic aetiology. In this population-based case-cohort study, we, for the first time, investigated variants of postulated hydrocephalus candidate genes. Using these data, we aimed to investigate potential involvement of the ciliome in hydrocephalus and describe genotype–phenotype associations with an autism spectrum disorder. One-hundred and twenty-one hydrocephalus candidate genes were screened in a whole-exome-sequenced sub-cohort of the Lundbeck Foundation Initiative for Integrative Psychiatric Research study, comprising 72 hydrocephalus patients and 4181 background population controls. Candidate genes containing high-impact variants of interest were systematically evaluated for their involvement in ciliary function and an autism spectrum disorder. The median age at diagnosis for the hydrocephalus patients was 0 years (range 0–27 years), the median age at analysis was 22 years (11–35 years), and 70.5% were males. The median age for controls was 18 years (range 11–26 years) and 53.3% were males. Fifty-two putative hydrocephalus-associated variants in 34 genes were identified in 42 patients (58.3%). In hydrocephalus cases, we found increased, but not significant, enrichment of high-impact protein altering variants (odds ratio 1.51, 95% confidence interval 0.92–2.51, *P = 0.096*), which was driven by a significant enrichment of rare protein truncating variants (odds ratio 2.71, 95% confidence interval 1.17–5.58, *P = 0.011*). Fourteen of the genes with high-impact variants are part of the ciliome, whereas another six genes affect cilia-dependent processes during neurogenesis. Furthermore, 15 of the 34 genes with high-impact variants and three of eight genes with protein truncating variants were associated with an autism spectrum disorder. Because symptoms of other diseases may be neglected or masked by the hydrocephalus-associated symptoms, we suggest that patients with congenital hydrocephalus undergo clinical genetic assessment with respect to ciliopathies and an autism spectrum disorder. Our results point to the significance of hydrocephalus as a ciliary disease in some cases. Future studies in brain ciliopathies may not only reveal new insights into hydrocephalus but also, brain disease in the broadest sense, given the essential role of cilia in neurodevelopment.

## Teaser

Munch *et al.* report 121 hydrocephalus candidate genes were screened in a whole-exome-sequenced cohort comprising 72 hydrocephalus patients and 4181 background population controls. The results 11 point to the significance of hydrocephalus as a ciliary disease in many cases, which may explain the frequent co-occurrence of other brain disease.

## Graphical Abstract

**Graphical abstract:**
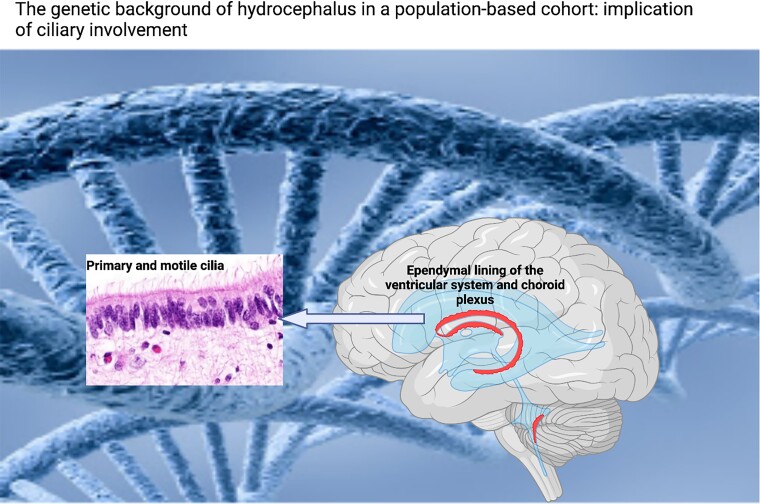

## Introduction

Hydrocephalus is a complex and heterogenic condition in which the volume of intracranial CSF is excessive relative to the brain volume due to different mechanisms encompassing (i) structural blockage of CSF circulation^1^; (ii) altered permeability of the ependymal lining of the ventricular system and choroid plexus^2^; and (iii) impaired neurodevelopment.^3^ At the same time, it is one of the most common congenital disorders of the central nervous system affecting 1.08 in 1000 live-born children, when all subtypes are included.^4^ At the time of writing, 100+ candidate genes for hydrocephalus have been described in referred patients and animal studies,^5–8^ including eight genes known to exhibit a Mendelian pattern of inheritance, namely, two X-linked genes: *L1CAM* and *AP1S2*, and six autosomal genes; *CCDC88C, MPDZ, TRIM71, SMARCC1, PTCH1* and *SHH.*^5–9^ There are no findings from genome-wide association studies, which is probably due to difficulties to identify sufficient case numbers to identify moderate or small effect sizes, and the situation is further complicated by the heterogeneous presentations of hydrocephalus. Several of the aforementioned genes are known to play important roles in early brain development, whereas others lead to impaired CSF dynamics, but overall, the understanding of the actual disease mechanisms leading to hydrocephalus at a molecular genetic level is still in a burgeoning stage.^6,9–11^

Emerging evidence suggests a critical function of cilia in neurodevelopmental and psychiatric disorders.^12–15^ Cilia comprise a diverse group of antenna-like and microtubules-based organelles, which project from centrosomes or centrioles at the cell surface and play key roles in cell motility and/or signalling, in turn controlling development and function of many types of tissue and organs in our body.^14,16^

Cilia in the brain have functional diversities and therefore ciliopathies may, by different mechanisms, lead to hydrocephalus.^17^ Motile cilia in the ependymal lining of the ventricular system conduct the wall-near CSF flow and together with the pulse generated bulk flow ensures circulation between the compartments of the ventricular system and further the flow through the outlets of the fourth ventricle to the subarachnoid space and further facilitating the circulation to the venous sites of CSF absorption.^18,19^ Motile ciliopathies can lead to hydrocephalus due to impaired circulation and subsequent accumulation of CSF.^20,21^ Furthermore, motile ciliopathies may impair the transport of signalling molecules and cells necessary for normal neurodevelopment.^19^ Another suggested mechanism is that decreased CSF flow impairs the patency of the different outlets of the ventricular system thereby causing obstructive hydrocephalus due to, for instance, stenosis of the Sylvian aqueduct that connects the third and fourth ventricle.^22,23^

The immotile sensory/primary cilia in the ventricular lining interact with subventricular stem cells and play crucial roles in their activation, maturation and migration due to the roles of primary cilia in Wnt- and sonic hedgehog signalling.^13,24,25^ The impaired formation of the brain parenchyma and/or structural abnormalities of the ventricular system can lead to hydrocephalus as well as typically associated abnormalities such as dys- or agenesia of the corpus callosum, decreased cortical development, posterior fossa abnormalities and associated cystic formations.^24,25^ The choroid plexus cilia appear to only have transient motility during the perinatal period,^26,27^ whereafter they are involved in regulating ion transport and CSF production.^28^ Impaired function may lead to hydrocephalus due to overproduction of CSF but without impairment of neurodevelopment.^28–30^

Ciliary genes encode structural proteins in the cilium, proteins directly involved in ciliary function, including the signalling pathways compartmentalized into the ciliary organelle, or genes involved in maintaining ciliary function or structural integrity within the cell.^31,32^ Understanding the genetic disease mechanisms leading to hydrocephalus may also provide an explanation of the strong association between hydrocephalus and autism spectrum disorder that has been found in another sub-study of the Lundbeck Foundation Initiative for Integrative Psychiatric Research (iPSYCH) cohort,^33^ as well as in other populations.^34–36^ Access to the iPSYCH cohort data enabled us to conduct this population-based case-cohort study investigating virtually all postulated hydrocephalus candidate genes (121) in 72 whole-exome-sequenced hydrocephalus cases for putatively hydrocephalus-causing variants, compared with 4181 exome-sequenced background population controls without psychiatric disorders, hydrocephalus or any of the neurologic conditions preselected by the iPSYCH group. Furthermore, we explored each of the genes with high-impact variants to understand the involved disease mechanisms with specific focus on potential involvement of the ciliome, and whether the involved genes previously had been associated with phenotypes with co-occurring autism spectrum disorder, based on the literature.

## Materials and methods

### Data sources

This cohort study was purely register-based and it is a sub-study of iPSYCH^37^ and combines data from The Danish National Patient Register,^38^ The Danish Neonatal Screening Biobank^39^ and the Danish Psychiatric Central Research Register.^40^ Linkage between registers and existing biobank data on whole-exome sequencing results was possible due to the unique personal identification number from the Danish Civil Registration System assigned to all individuals living in Denmark.^41^

### Source population: the iPSYCH cohort

All members of the iPSYCH cohort were born between 1981 and 2005 and the cohort was followed until 31 December 2016. The cohort members fulfilled two basic requirements: (i) being alive at 1 year of age, and (ii) the mother was residing in Denmark holding a Danish Civil Registration number [ Central Person Register (CPR)-number]. The CPR-number was used to link with the Danish National Patients Register as well as the Danish Psychiatric Central Register, in order to obtain information on diagnoses of the entire cohort. The Danish Civil Registration System covers the entire country, and it is continuously updated on vital status and migration.^42^ Reporting of diagnoses to the Danish National Patients and Psychiatric Central Register happens continuously and is mandatory for all public and private hospitals in Denmark. The cohort members were followed until one of the following events: migration, death or end of follow-up on 31 December 2016.

### Study population

**Figure 1:**
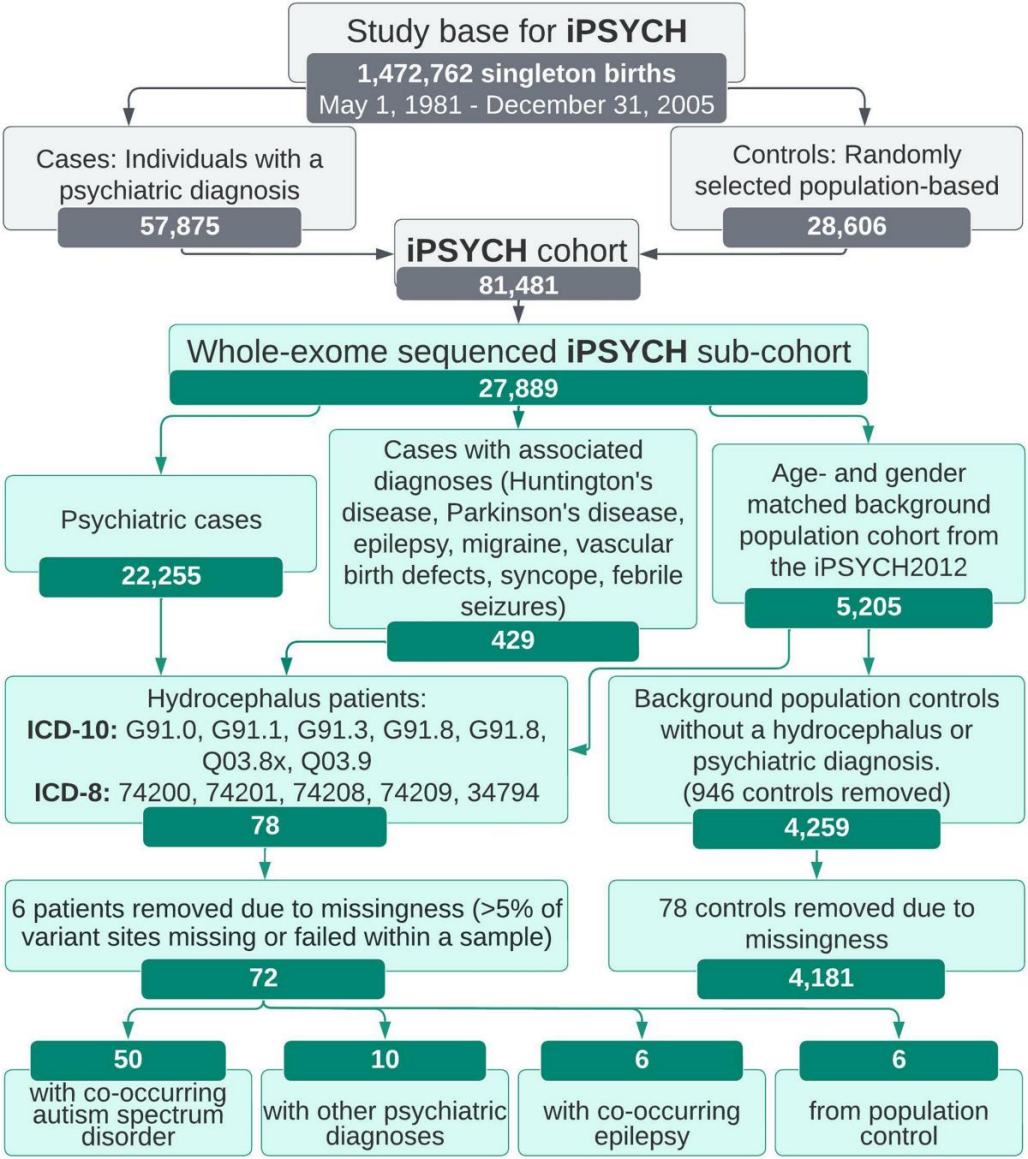
**Flow chart of the study population.** The upper four boxes briefly indicate the distribution of cases and controls from the original Lundbeck Foundation Initiative for Integrative Psychiatric Research (iPSYCH ) case-cohort sample. The boxes below indicate the distribution of cases and controls in the present study utilizing the exome sequenced data from iPSYCH. The associated diagnoses represent neurologic conditions which have been associated with psychiatric disease within the iPSYCH cohort. The limit of missingness was >5% of variant sites missing or failed within a sample. ICD, International Classification of Diseases.

Patients and controls were obtained from the iPSYCH cohort, which contains genetic and diagnostic information on 27 889 whole-exome sequenced Danes.^37^ Of these, 22 255 were cases who had received a psychiatric diagnosis, 429 had received a diagnosis with known psychiatric associations and 5205 background population controls were extracted from the randomly sampled iPSYCH population-based cohort.^37^ We identified 78 hydrocephalus cases and 4259 background population controls without psychiatric disorders, hydrocephalus or any of the neurological conditions preselected by the iPSYCH group (Huntington’s’ disease, Parkinson’s disease, epilepsy, migraine, birth defect in heart and major arteries or veins, syncope and febrile seizures) registered during the study period from the exome-sequenced population in the iPSYCH cohort. No relatedness or stratification assessments were performed. However, the mtDNA distributions in hydrocephalus cases in the cohort are identical to that found in controls^43^, suggesting that HC cases do not constitute a subfraction of the Danish population. Six hydrocephalus patients and 78 controls were removed from the analysis due to missingness (samples where >5% of variant sites were missing or failed). Out of the 72 remaining hydrocephalus cases, 50 also had a registered diagnosis of autism spectrum disorder, 10 were diagnosed with other psychiatric disorders, 6 were diagnosed with epilepsy and 6 individuals were from the background population. The hydrocephalus cases were compared with the remaining 4181 background population controls. Figure 1 illustrates the selection steps of the study population.

### Ascertainment of hydrocephalus

The study base was linked, through the CPR-number, to the National Patient Register. Hydrocephalus cases were identified using the following hydrocephalus diagnoses classified according to the two different versions of the International Classification of Diseases (ICD) used during the study period. ICD-8: 74200, 74201, 74208, 74209 and 34794. ICD-10: G91.0, G91.1, G91.3, G91.8, G91.9, Q038x and Q039.

### Selection and analysis of candidate genes

From the literature, we identified 121 genes where mutations had previously been implicated in hydrocephalus or were part of molecular pathways known to harbour hydrocephalus-causing genes^6,8^ (Supplementary Table 1). Analyses of the 121 candidate genes were performed on the exomes from both cases and controls. DNA extraction and exome sequencing from dried blood spots have been described previously for this cohort.^37,44^

### Quality control assessments

Filtering steps were adapted from Satterstrom *et al.*^45^ Briefly, all variants were filtered as follows: (i) variants with a GATK variant quality score recalibration label of ‘PASS’ and a depth of coverage between 10 and 1000 were selected. (ii) low-quality variants were removed; these variants were defined as (a) homozygous reference calls with a GQ < 25 or fewer than 90% of reads supporting the reference allele call. (b) Homozygous variant calls with a PL (HomRef) < 25 or fewer than 90% of reads supporting the alternate allele call. (c) Heterozygote calls with a PL (HomRef) < 25, fewer than 25% reads supporting the alternate allele, fewer than 90% informative reads, a probability of drawing the allele balance from a binomial distribution centred on 0.5 of <1e−9, or a location where the sample should be hemizygous (e.g. calls on the X chromosome outside the pseudo-autosomal region in a male). (d) Y-chromosome calls (outside the pseudo-autosomal region) in a female. (iii) Variants with a call rate below 80% were removed.

As the sequence alignment map format was not accessible due to iPSYCH’s adherence to the American College of Medical Genetics recommendations for reporting incidental findings,^46^ indirect estimations of gene-specific exon sequencing coverage were performed using allele frequency (AF) from gnomAD v2.1.1 and the study population. Briefly, the gnomAD minor AF (MAF) for variants identified in samples of Non-Finnish European ancestry in the gnomAD controls subset (controls_AF_nfe) were compared with the MAF in variants identified in the study population. They were found to be highly correlated (*r*^2^ = >0.977, *P* = 0) for AF > 0.002. The number of expected identified variants per gene was defined as exon variants in gnomAD with controls_AF_nfe ≥ 0.002 [Corresponding to ∼13 alleles after adjusting for minimum accepted genotype call rate (0.8)]. The probability of not-having an observation at an included site was estimated to be *P <* 9.2 × 10^−7^. The indirect estimation of gene region coverage is shown in Supplementary Table 2.

Variants were annotated using SnpEff GRCh37.p13.RefSeq using only protein coding transcripts and CADD v.1.4. All conflicting variant annotations according to GRCh37.p13.RefSeq were excluded. A total of 5854 variants remained after QC.

As an estimate of case/control comparability, the proportions of synonymous alleles (allelic-load) in cases and controls were compared using two proportions *z*-test with and without adjusting for missing genotype rate. For the non-adjusted, the proportions were 3.2% for cases and 3.15% for controls (*P* = 0.12) and the adjusted 3.2% and 3.2% (*P* = 0.13), respectively.

### Prediction and classification of high-impact variants

To discriminate between high- and low-impact variants, we used CADD v.1.4 PHRED scored variants in the candidate genes. Variants reported in ClinVar (clinvar_20210814)^47^ and gnomAD (v2.1.1) were used to find the best performing (f1-score) PHRED score cut-off for each of the sequence ontology classes (stop-gained, stop-lost, splice-site, non-synonymous, in-frame, frame-shift and canonical-splice). Low-impact variants were defined as: variants reported in ClinVar in which the review status was reported as either: ‘reviewed_expert_panel’, ‘criteria_provided, multiple_submitters, no_conflicts’ or ‘criteria_provided, single_submitter’ and the clinical significance was reported as either: ‘benign’, ‘likely_benign’, in addition to gnomAD (v2.1.1) variants with MAF in all samples or controls_AF_nfe >0.001 and not present in ClinVar. High-impact variants were defined as: variants reported in ClinVar in which the review status was reported as either: ‘reviewed_expert_panel’, ‘criteria_provided, multiple_submitters, no_conflicts’ or ‘criteria_provided, single_submitter’ and the clinical significance was reported as ‘pathogenic’.

For sequence ontology classes: non-synonymous, splice-site and in-frame the PHRED cut-offs were found using a classification and regression trees algorithm (rpart v.4.1–15). For sequence ontology classes: canonical-splice, frame-shift, stop-gained and stop-lost, too few low-impact variants were identified; consequently, all variants of these classes were classified as high impact. The performance and methods used for the high-/low-impact classification of each sequence ontology class are shown in Supplementary Table 3. Additionally, predicted high-impact variants were reclassified as low impact if the MAF in controls_AF_nfe from gnomAD (v2.1.1) exceeded 0.001 and variants present in ClinVar were reclassified according to their clinical significance status. Comparing cases with controls, there was a statistically significant difference in being called missing or failed in 0.8% of the high-impact variants.

### Sensitivity analysis

To assess the uncertainty of the chosen CADD PHRED and AF cut-offs, several sensitivity analyses were performed for non-synonymous, splice-site, in-frame and protein truncating variants (PTVs) (defined as canonical-splice, frame-shift, stop-lost, stop-gained). AF ranging from the minimum observed in the study population frequency (0.0001) to 0.1 (non-synonymous variants) and 0.01 (splice-site variants, in-frame variants and PTVs) were assessed in 0.0005 intervals (Supplementary Table 3). For each AF cut-off, the high-impact variant allele load, adjusted for missing genotype rate, was calculated and compared for cases and controls using Fisher’s exact test (Supplementary Table 4). Since all in-frame and splice-site variants occurred in control samples, the CADD PHRED cut-offs were assessed in non-synonymous variants only. PHRED range from 11 to 31.8 in 0.2325 intervals.

### Statistical analysis

To assess the uncertainty of the chosen CADD PHRED and AF cut-offs, a sensitivity analysis was performed for high-impact variants, as described above. For each PHRED cut-off, the high-impact variant allele load, adjusted for missing genotype rate, was calculated and compared for cases and controls using Fisher’s exact test (Supplementary Table 5). The difference between the number of carriers of high-impact variants among cases and controls was tested using Fisher’s exact test. Subsequent analyses tested the variant categories PTV and non-PTV. Student’s t-test and the Wilcoxon test were used for comparison of missingness per sample between cases and controls. All statistical analyses were performed in R 3.6.1.

### Phenotypes and mechanisms of disease

The pathogenicity of the candidate genes with high-impact variants was investigated using The Human Gene Mutation Database,^48^ Online Mendelian Inheritance in Man^49^ and CiliaCarta.^32^ Protein structure/function consequences and evolutionary conservation of all variants were assessed by mutation taster.^50^ Furthermore, we conducted a systematic literature search in order to detect hydrocephalus phenotypes not yet described in the gene databases and to investigate the role of the gene in ciliary function. Thus, PubMed was searched for the name of each of the screen-positive genes and/or the protein in combination with ‘hydrocephalus’, ‘ventriculomegaly’, ‘cilia’, ‘ciliopathies’, ‘autism’ and ‘ASD’.

### Patient consent

The study is a sub-study of the iPSYCH study, that was approved by the Scientific Ethics Committees of the Central Denmark Region (www.komite.rm.dk) (J.nr.: 1–10-72-287-12) and the Danish Data Protection Agency (https://www.datatilsynet.dk/) (J.nr.: 2012-41-0110). Passive consent was obtained, in accordance with Danish Law nr. 593 of 14 June 2011, para 10. The Danish Neonatal Screening Biobank approved use of the dried blood spot samples stored in the Danish Neonatal Screening Biobank. Detailed information on governance and ethics in the iPSYCH cohort is available at the iPSYCH website (www.ipsych.au.dk).

## Results

Among the 27 889 whole-exome-sequenced individuals, we identified 78 patients diagnosed with hydrocephalus. Following quality control assessment, samples with missing genotype call *Z*-score> 3 or < −3 were excluded, resulting in six hydrocephalus patients and 78 background population controls were removed, leaving 72 hydrocephalus cases and 4181 controls (Fig. 1). Of the remaining, included samples the missingness per sample was not statistically significant between cases and controls (*P = 0.44/0.17* t-test/Wilcoxon). The percentage of variants with statistically significant (*P* < 0.05) case/control missingness rate was 1.6%. The median age at diagnosis for the hydrocephalus patients was 0 years (range 0–27 years), the median age at analysis was 22 years (11–35 years) and 70.5% were males. The median age for controls was 18 years (range 11–26 years) and 53.3% were males. Screening of the exomes for 121 previously reported hydrocephalus candidate genes resulted in the identification of 52 high-impact variants in 34 susceptibility genes, which are presented in Supplementary Table 6. Of these, eight genes were carrying a total of nine PTVs: *B3GALNT2, CELSR2, CENPF, DAG1, IFT172, MTO1, NF1 (two variants)* and *PTCH1.* All but *MTO1* genes were found to be involved in ciliary function, according to the literature. Detailed descriptions of all hydrocephalus-associated genes with high-impact variants and their involvement in ciliary function are described in the Supplementary material, as well as the overall gene function, phenotypes and associations with ASD.

### Distribution of variants and burden analysis

Testing the carrier status for high-impact variants over all 121 genes of interest between hydrocephalus cases and controls showed an increased but not significant number of high-impact variants among cases (OR 1.51, 95% CI 0.92–2.51, *P = 0.096*). Subsequent analysis of the variant sub-groups PTVs and non-PTVs showed that the increase was mainly due to PTVs (OR 2.71, 95% CI 1.17–5.58, *P = 0.011*, Table 1). Furthermore, we performed an analysis of the occurrence (and type) of high-impact variants in 121 genes in the 6232 patients with an autism spectrum disorder in the iPSYCH cohort. The results show that the controls and patients with autism did not differ in variant distribution.

**Table 1:** Distribution of high-impact variants and PTVs among the 121 genes screened in 72 hydrocephalus cases, 4181 background population controls and 6232 ASD cases

Variant type	Carriers by # hits	Cases, *n* (%)	Controls, *n* (%)	*P*-value	OR	95% CI	ASD, *n* (%)
High impact	All carriers	42 (58.3)	2009 (48.1)	0.096	1.51	0.92–2.51	3060 (49.1)
High impact	1	32 (44.4)	1425 (34.1)				2154 (34.6)
High impact	2	8 (11.1)	456 (10.9)				702 (11.3)
High impact	3	2 (2.8)	100 (2.4)				170 (2.7)
High impact	4	0	24 (0.6)				28 (0.4)
High impact	5	0	4 (0.1)				5 (0.1)
PTVs	All carriers	9 (12.5)	209 (5.0)	0.011	2.71	1.17–5.58	337 (5.4)
Non-PTVs	All carriers	33 (44)	1800 (43.1)	0.72	1.12	0.68–1.83	2723 (43.7)
PTVs	1	9 (12.5)	200 (4.8)				330 (5.4)
PTVs	2	0	9 (0.2)				6 (0.1)
PTVs	3	0	0				1 (0.0)

The results of burden tests are shown for high-impact variants in total, as well as for PTV- and non-PTV-high-impact variants.

A detailed description of the distribution and types of variants found in the subset of 34 genes with high-impact variants in hydrocephalus cases is given in Supplementary Tables 7 and 8. No homozygote carriers and two hemizygote carriers were identified among hydrocephalus cases, whereas one and four carriers, respectively, were identified among controls. Of the 52 high-impact variants carried by 42 of the hydrocephalus patients, 9 were PTVs, of which 2 were canonical splice site variants, 4 were frame-shift variants and 3 were stop-gained variants. All 43 non-PTVs were non-synonymous variants. As expected, given that the subset of genes is selected on high-impact variants in hydrocephalus cases, higher percentages were observed in the hydrocephalus cases.

### Genes with ciliary function according to the literature

According to CiliaCarta^32^ and a literature search on each of the genes containing high-impact variants, we found that 14 of the 34 genes carrying high-impact variants had a well-established ciliary function and another 6 genes were involved in cilia-dependent processes in neuronal formation. Among the eight genes carrying PTVs, we found that four genes have well-described ciliary functions: *CELSR2* (primary and motile cilia),^51–53^*CENPF* (ciliogenesis, microtubule dynamics),^32,54^*IFT172* (primary and motile cilia, intraflagellar transport)^32,55^ and *PTCH1* (primary and motile cilia).^5,56^ Another three genes carrying PTVs were found to be involved in processes regulated by primary cilia in the brain: *B3GALNT2, DAG1* and *NF1.*

*B3GALNT2* encodes Beta-1,3-*N*-Acetylgalactosaminyltransferase 2, which is involved in the glycosylation of alpha-dystroglycan (α-DG). Reduced glycosylation impairs the interaction of α-DG with extracellular matrix elements,^57^ which may disrupt the signalling functions of primary cilia, and lead to brain malformations.^58^*DAG1* which encodes dystrophin-associated glycoprotein 1, which forms part of the dystrophin-associated protein complex consisting of dystroglycans, sarcoglycans, dystrobrevins and syntrophins. Dystrophin-associated protein complex components are expressed and regulated during the neuronal or astrocytic differentiation of neural stem/progenitor cells.^11^*NF1* which encodes neurofibromin 1, a tumour suppressor which is expressed largely in neuronal cells, it is known to associate with microtubules and is involved in several signalling pathways.^59^ The neurofibromatosis changes may link to the function of primary cilia, as these are required for the formation of neural stem cells (NSCs) located in the subventricular zones, primarily of the lateral ventricles in adults and the third ventricle and optic pathway in children as well as for neurogenesis through Hedgehog signalling.^60–62^

The remaining 11 genes with well-defined ciliary function carrying high-impact variants (non-PTVs) encompassed: *DNAH5* (motile cilia),^32,63^*DNAI1* (motile cilia),^64,65^*FLNA* (ciliogenesis),^32,66^*FUZ* (ciliogenesis, primary and motile cilia),^67–69^*LRP 6* (primary cilia),^70,71^*MPDZ* (ciliogenesis, motile cilia),^72^*NOTCH2* (primary cilia),^73,74^*PIK3R2* (primary cilia, ciliogenesis),^75^*SMARCC1* (primary cilia),^76,77^*TRIM71*(motile cilia)^78^ and *VANGL2* (primary and motile cilia, ciliogenesis).^32,52,79^ In Table 2, all the high-impact variant carrying genes with ciliary involvement are described and the genes with PTVs are underlined.

**Table 2:** The subsets of 14 genes with ciliary function and the six genes with involvement in cilia-dependent processes, according to CiliaCarta and/or the literature among the 34 candidate genes associated with hydrocephalus

	CiliaCarta score^a^	CiliaCarta status^b^	Ciliary function^c^
**Genes with ciliary function**			
***CELSR2***	−5.503	G	Development and planar organization of ependymal motile cilia
***CENPF***	−4.235	N	Centromer protein F. CENPF protein is involved in early ciliogenesis mainly in basal body (mother centriole) development
***DNAI1***	7.043	E	Structure and function of the motile ependymal cilia compartmentalized to the outer dynein arm
***DNAH5***	2.268	E	Structure and function of the motile ependymal cilia compartmentalized to the outer dynein arm
***FLNA***	−1.636	E	Ciliogenesis and basal body positioning
***FUZ***	0.8617	E	Development and planar organization of ependymal motile cilia
***IFT172***	9.287	E	Intraflagellar transport protein required for ciliogenesis and hedgehog signalling
***LRP6***	−7.292	G	LRP 6 is a Wnt co-receptor, Cilia-dependent Wnt signalling is important for neurogenesis
***MPDZ***	1.05	G	Involved in PCP, which coordinates the cilia beating and directs CSF circulation
***NOTCH2***	−5.900	N	Primary cilia have been linked to Notch signalling pathways, which affect cell proliferation and migration. NOTCH2 has been associated with choroid plexus tumours
***PIK3R2***	−2.503	G	GSK-3β kinase is involved in the formation of centrosome-derived microtubules of the primary cilia and inhibition of GSK-3β results in reduced occurrence of primary cilia
***PTCH1***	−1.494	E	Encodes Sonic hedgehog receptor on the primary cilium, neural tube development and regulation of ventricular zone neural stem cell fate. Furthermore, functionally compromised motile cilia
***TRIM71***	−6.383	G	Neural tube development and regulate ventricular zone neural stem cell fate
***VANGL2***	−6.304	E	Disrupted rotational and translational PCP of the ependyma cells leading to disrupted beating of motile cilia
**Genes with involvement in cilia-dependent processes**			
***ASTN2***	−0.859	G	Encodes astrotactin 2, which is involved in neuronal migration, a process regulated by primary cilia
***B3GALNT2***	−3.507	G	Encodes a glycosyltransferase enzyme involved in α-DG glycosylation. α-DG is a cell surface receptor for extracellular matrix proteins and dysfunction may, therefore, disrupt the signalling functions of primary cilia
***DAG1***	−5.238	G	The dystrophin-associated protein complex components are expressed and regulated during the neuronal or astrocytic differentiation of neural stem/progenitor cells; a process regulated by primary cilia
***NF1***	−1.64	G	Primary cilia are required for the formation and activation of the neuronal stem cells, which in neurofibromatosis leads to the formation of optic gliomas and chiasmatic-hypothalamic tumours
***ROBO1***	−4.882	G	Interact with notch signalling through Hes1 expression in ventricular zone progenitors and thereby links with primary cilia function
***SMARCC1***	−4.913	G	Regulates gene expression required for neural stem cell proliferation, differentiation and survival during telencephalon development, processes involving primary cilia-dependent signalling

a The CiliaCarta Score is a log-transformed odds which means that a positive score indicates that a gene is more likely to be ciliary than non-ciliary.

b If a gene belonged to the positive training set of established ciliary genes (E), the negative training set of genes with unlikely ciliary function (N) or predictions for novel ciliary genes (G). Notably, a negative CiliaCarta score or N do not exclude ciliary function, if described in the literature.

c Cilia function was established by literature search and CiliaCarta status. References and descriptions can be found in the Supplementary material.

Gene carrying PTVs have been underlined.

### Genes associated with autism spectrum disorder

For 15 of the 34 genes with high-impact variants observed in the hydrocephalus cases (*n* = 20 carriers), there are reports in the literature for involvement in ASD. Six patients carried PTVs in three of these genes (*PTCH1, DAG1* and *NF1). PTCH1* is part of the ciliome, and *DAG1* and *NF1* are involved in cilia-dependent processes of neurogenesis. In total, 13 of the 15 high-impact variants (including all three PTVs) were observed in patients with co-occurring ASD diagnosis. Proposed mechanisms of disease are summarized in Table 3 for the involved genes and elaborated in the Supplementary material.

**Table 3:** Overview of the 15 hydrocephalus genes with high-impact variants carried by 20 patients, which were previously reported to be involved in ASD, according to phenotype descriptions in the literature.

	Gene function	Suggested mechanism	Psychiatric comorbidity in carriers (*n* = 20)
***ASTN2* ^a^**	Encodes astrotactin 2, which is involved in neuronal migration, and is considered an ASD susceptibility gene		ASD + affective disorder in two different carriers
***DAG1* ^a^**	Dystrophin-associated proteins complex are expressed and regulated during the neuronal or astrocytic differentiation of neural stem/progenitor cells	Impaired neuronal or astrocytic differentiation	ASD + affective disorder in one carrier and schizophrenia in another
***FLNA* ^b^**	Filamin A has a crucial role in ciliogenesis and basal body positioning	Arrest of neuronal migration	ASD in one carrier
***LAMB1***	Encodes laminin, beta 1, an extracellular matrix protein	Impaired axonal outgrowth and guidance	ASD in one carrier
***MPDZ* ^b^**	Encodes multiple PDZ domain protein 1 (MUPP1), which is important for synaptic adhesion. Involved in PCP	Impaired CASPR2–MUPP1–GPR37 complex on the dendrites is associated with one of the pathogeneses of ASD	ASD + affective disorder in one carrier
***NF1* ^a^**	Formation of NSCs located in the subventricular zones, primarily of the lateral ventricles in adults and the third ventricle and optic pathway in children	Impaired neurogenesis through hedgehog signalling	ASD in one carrier and ASD + ADHD in another
***NOTCH2* ^b^**	Encodes the notch receptor 2 and is thereby essential for notch signalling	Impaired cortical neurogenesis	ASD + ADHD in one carrier
***PIK3R2* ^b^**	Regulatory subunit of the PI3K–AKT–mTOR pathway, modulates GSK-3β activity	Associated with brain overgrowth. GSK-3β kinase is involved in the formation of centrosome-derived microtubules of the primary cilia and inhibition of GSK-3β results in reduced occurrence of primary cilia	ASD + affective disorder in one carrier
***PLOD2***	Regulates the expression of downstream epithelial–mesenchymal transition-associated regulators in the PI3K/AKT signalling pathway.	ASD, together with skeletal abnormalities, has been associated with *PLOD2* mutations	ASD + affective disorder in one carrier
***POMT1***	Encodes protein-*O*-mannosyltransferase 1, essential for α-DG glycosylation	Impaired neuronal migration	Affective disorder + schizophrenia in one carrier
***PTCH1* ^b^**	Encodes patched 1, the Sonic hedgehog receptor	Impaired neural tube development and regulation of ventricular zone neural stem cell fate	ASD + affective disorder in one carrier, ASD in another
***ROBO1* ^a^**	Regulates Hes1, a major repressor of Notch signalling.	*ROBO1* mutations interfere with early neural differentiation	ASD in one carrier and ADHD in another
***SHOC2***	Encodes leucine-rich repeat scaffold protein, which is part of the RAS/MAPK pathway	Impaired neurogenesis	ASD + ADHD in one carrier
***SMARCC1* ^a^**	Encodes an ATP-dependent chromatin remodeller that regulates gene expression required for neural stem cell proliferation	Impaired neural stem cell proliferation, differentiation and survival during telencephalon development	Background population control
***VANGL2* ^b^**	Neural tube closure, PCP signalling and beating of motile ependymal cilia. Involved in Wnt signalling	Possibly affected the interaction between Vangl2 and another PCP protein, Prickle 2	Background population control

**Abbreviations:** ADHD = Attention Deficit and Hyperactivity Disorder; Akt (PKB) = Protein Kinase B; ASD = Autism Spectrum Disorder; ATP = Adenosine triphosphate; CADD = Combined Annotation Dependent Depletion; CENPF = Centromere Protein F; CI = confidence interval; CNS = Central nervous system; CSF = Cerebrospinal fluid; GATK = The Genome Analysis Toolkit; GQ = Gq protein alpha subunit; GSK = Glycogen synthase kinase-3 beta; HC = Hydrocephalus; LRP = Low Density Lipoprotein Receptor Related Protein; MAPK = Mitogen-Activated Protein Kinase; PHRED = Phil's Read Editor; PL = Phred-scaled likelihoods; RAS = Rat Sarcoma Virus; mTOR = Mammalian Target of Rapamycin; CASPR = Contactin Associated Protein.

a Genes involved in cilia-dependent processes of neurogenesis (*n* = 5).

b Belongs to the ciliome (*n* = 6).

Genes carrying PTVs have been underlined.

## Discussion

In this population-based case-cohort study encompassing 72 hydrocephalus patients and 4181 background population controls from the same birth cohort, whole-exome sequences from both cases and controls were screened for a panel of virtually all known candidate genes for hydrocephalus. Of the 121 investigated candidate genes, we identified 52 high-impact variants in 34 of the genes, carried by 42 of the hydrocephalus patients (58%). The number of high-impact variants over all 121 genes of interest between hydrocephalus cases and controls was higher in hydrocephalus cases albeit not significant (OR 1.51, 95% CI 0.92–2.51, *P = 0.096*), whereas hydrocephalus cases were significantly enriched with the variant subgroup PTV’s (OR 2.71, 95% CI 1.17–5.58, *P = 0.011*). Interestingly, 20 of the 34 genes with high-impact variants (59%), and seven of the eight (88%) hydrocephalus genes with PTVs, are either well-described parts of the ciliome or are involved in cilia-dependent processes in neurogenesis. The heterogeneity of the high-impact variants indicates that the association with hydrocephalus is not caused by relatedness.

### Hydrocephalus as a ciliopathy

The last two decades have seen a rapid increase in the number of genes involved in ciliogenesis and ciliary function, at the time of writing 302 genes with confirmed ciliary function according to CiliaCarta,^32^ with many more reported in the literature. The functions of both primary and motile cilia are dependent upon interaction of several functional compartments in the organelle. Thus, elements in the basal body and transitional zone seem to be involved in pathways controlling more distal ciliary functioning.^13,14,16^ This also supports that modification of the function in a gene in one component may have significant effects on ciliary motor or sensory function. A ciliary function is very complex with function controlled by cytoplasmic signals as well as direct signals to specific locations on the cilium, e.g. sonic hedgehog signalling through receptors on the ‘tip’ of the primary cilium.^80,81^ Furthermore, Wnt/β-catenin^70,71^ and Notch signalling^82^ involves interaction with cilia.

Mutations in *CELSR2, PTCH1* and *MPDZ* have been shown to impair ependymal flow, which in itself is believed to be one of the mechanisms of hydrocephalus formation.^23^ Furthermore, MRI-based computational fluid dynamic simulation in a human demonstrated that wall-near CSF flow is controlled by motility of ependymal cilia, whereas the flow in the centre of the third ventricle and Sylvian aqueduct are the result of macro-scale ventricular wall motion and choroid plexus pulsation.^18^ The direction of the CSF flow is determining for the direction of neuroblast migration, and thereby the organization of the brain tissue.^83^ Thus, deficient CSF flow may explain the co-existence of neurodevelopmental disorders and other brain pathologies in hydrocephalus. However, most of the screen-positive ciliary genes relate to the function of the primary cilium, which, by other mechanisms, also is crucial for neurodevelopment.^12,15,16^ Several of the screen-positive genes affect the three major signalling systems, Wnt/β-catenin, Sonic Hedgehog and notch signalling, which are associated with or compartmentalized to primary cilia.^16,24,84,85^

*SMARCC1* and *TRIM 71* are two hydrocephalus-associated genes with well-described phenotypes and involvement in primary and motile ciliary function, respectively.^5,8,78^ Whereas *SMARCC1* mutations cause aqueductal stenosis and with white matter volume reduction, as well as cardiac and skeletal abnormalities, patients with *TRIM71* mutations were prone to having to communicate hydrocephalus with insufficient development of ependymal cells with motile cilia in the lining of the ventricular system.^5,8^ A study in zebrafish revealed interaction between Smarcc1a and BBS6, which is involved in Bardet–Biedl syndrome and cardiac malformations.^86^ Bardet–Biedl syndrome serves as a study example of how several ciliary functions are affected; Bardet–Biedl syndrome mouse models involve ciliary intraflagellar transport, cilia maintenance, protein trafficking and regulation of CSF production.^87,88^ Corpus callosum abnormalities, complete septal agenesis or septal abnormalities, cerebellar tonsillar ectopia, developmental delay, epilepsy and malformations outside the CNS were additional common features in both *TRIM71* and *SMARCC1* mutation carriers.^8^ This illustrates the importance of both primary and motile cilia in neurogenesis. Rodríguez *et al.* suggest that the site of disrupted neurogenesis is of importance for the phenotype. The ventricular zone contains the multipotent radial glia/NSCs and the subventricular zone contains the rapidly proliferative neural precursor cells. According to Rodríguez *et al.*,^89^ disruption of the ventricular zone of the Sylvian aqueduct leads to aqueductal stenosis, while disruption of the ventricular zone of telencephalon impairs neurogenesis.

*CELSR2* and *MPDZ* are involved in planar cell polarity (PCP), which together with intercellular junctional complexes, form the tissue structure and coordinated processes across epithelial sheets. In the ependymal cells with multiple cilia, rotational and translational PCP coordinate the cilia beating and direct CSF circulation.^90^ Thus, PCP is essential for ciliary function and disruption results in ciliopathies and hydrocephalus.^53,90,91^ Studies of Mpdz-deficient mice showed that expression of the interacting PCP protein Pals1 was diminished and barrier integrity got progressively lost leading to enhanced permeability of the choroid plexus.^30,72,92^ According to Feldner *et al.*, the subsequent ependymal denudation was accompanied by reactive astrogliosis, which caused hydrocephalus due to aqueductal stenosis.^72^

The primary cilium is involved in integrating signals from the extracellular matrix and these interactions are significant for neuronal migration.^93^ Defective interaction with the extracellular matrix also interferes with Sonic Hedgehog and Notch signalling.^94,95^ Therefore, several of the genes with PTVs may indirectly affect cilia-dependent processes in neurogenesis: *B3GALNT2* encodes an enzyme essential for the correct glycosylation of α-DG, which impairs the interaction of α-DG with extracellular matrix elements,^57,96–99^ possibly disrupting the signalling functions of primary cilia. The genes *DAG1* and *NF1* are involved in the regulation of neuronal or astrocytic differentiation of neural stem/progenitor cells, processes regulated by primary cilia.^11,61,62^

### Co-occurring autism spectrum disorder

In total, 15 genes with high-impact variants (including all three PTVs) were previously associated with hydrocephalus-autism spectrum disorder phenotypes in the literature, as described in the Supplementary material. For 12 of these genes, we found that the carriers had co-occurring autism spectrum disorder. Thus, one could ask if our genetic findings simply reflected the high occurrence of autism spectrum disorder among the hydrocephalus cases (69%). According to our results (Table 1), the burden of hydrocephalus-associated high-impact variants among patients with autism spectrum disorder was not different from controls. Hence, the presence of autism spectrum disorder does not influence the aetiology of hydrocephalus. As presented in Table 3, hydrocephalus and neurodevelopmental disorders may share mechanisms of disease through similarly affected signalling pathways. Disruption of neuronal migration and differentiation is a key element in the aetiology behind neurodevelopmental disorders,^100,101^ processes, which per above, to a large extend involves primary cilia.

### Limitations and strengths

A limitation of the present study is that detailed clinical information was unavailable. It would have been very informative to be able to describe somatic co-morbidities, as cilia-related diseases are characterized by a very broad range of syndromic and non-syndromic phenotypes. Some of these manifestations, such as recurring upper airway infections and otitis media, are likely treated outside the hospital system, and therefore these co-diagnoses will not be registered in the Danish National Patients Register. Unfortunately, cilia-associated diseases have no distinct ICD-codes, not even primary ciliary dyskinesia.

Another limitation is that the study only includes individuals that were alive at 1 year of age, which may have led to an underestimation of hydrocephalus. In the Danish population, children with infantile congenital/hydrocephalus have a cumulative mortality of 20% at 4 years of age, compared with a mortality of 0.7% among children from the background population.^4^ Furthermore, this study did not include elective termination of pregnancies due to prenatal diagnosis of hydrocephalus, as abortions are not registered by diagnosis in Denmark. This differential mortality may have led to exclusion of hydrocephalus subtypes with early and severe presentations.

The availability of trios would have improved interpretation of our findings with respect to causality. We observed an overrepresentation of males (70.5%) among the cases compared with the background population controls (53.3%), supposedly explained by the overrepresentation of males in hydrocephalus and autism spectrum disorder,^102,103^ which constitutes a potential confounder. Yet, X-linked genetic causes were not driving our findings as only one out of 34 genes, *FLNA*, were localized to the X chromosome. Ideally, the study would have been replicated in an independent set of sequenced individuals with hydrocephalus from the general population and an independent group of hydrocephalus patients with autism spectrum disorder, and we advocate for such studies in the future. Notably, this is the first population-based study in which we investigated the presence of virtually all postulated hydrocephalus candidate genes, which is an important strength and improves the generalizability. Furthermore, all diagnoses were obtained from the Danish nationwide registries which boast complete national coverage.

### Clinical and research perspectives

The introduction of clinical genetics with respect to ciliopathies has the potential to become a game-changer in the personalized treatment of hydrocephalus. Advances in surgical treatment make highly individualized treatment possible, but failure rates remain extremely high (cumulative 1-year risks of 44–50%).^104,105^ By causing disturbed micro circulation, and in some cases increased protein levels in the CSF, ciliopathies may very well be the prevailing causes of these persistently high failure rates. If the presence of a ciliopathy is accounted for in the choice of surgical treatment, the failure rate may decrease. Future studies of associations between genetic profiles and the surgical outcomes could elucidate these hypotheses. For instance, a motile ciliopathy caused by *CELSR2, MPDZ* and *PTCH1* may be better alleviated by shunt treatment rather than endoscopic treatment, since the impaired CSF flow would maintain a ‘functional aqueductal stenosis’ after endoscopic treatment with third ventriculostomy. In the same line of thinking, mutations in *MPDZ* may help explain why some children are prone to shunt obstruction since mutations may lead to high permeability of the choroid plexus epithelial cell monolayer and thereby abnormally high CSF protein levels, which can cause shunt obstruction through accumulation of proteins in the ventricular catheter or shunt valve.^30^ Preoperative identification of *MPDZ* mutations may direct the treatment towards endoscopic surgical treatment, whenever possible and meaningful, or treatment with shunt systems less prone to obstruction.

Understanding the role of ciliopathies in hydrocephalus may furthermore pave the way for personalized medical treatment targeted at restoring ciliary function, as exemplified by the use of lentiviral gene therapy in Primary ciliary dyskinesia.^106^ Another example is the use of melanin-concentrating hormone that may increase the ciliary beating frequency.^107^ Moreover, downstream platelet-derived growth factor receptor activation by lithium in a Bardet–Biedl syndrome mouse model reduced the ventricular volume.^108^

## Conclusion

Our results support a significant genetic component of the aetiology of hydrocephalus and based on several lines of evidence, our results point to the significance of hydrocephalus as a ciliary disease. Furthermore, the results indicate overlapping molecular genetic aetiologies of hydrocephalus and autism spectrum disorder. We suggest clinical genetic assessment of congenital hydrocephalus patients with respect to ciliopathies and genetic predisposition for autism spectrum disorder as identification of co-morbidities may be of great clinical significance for the individual patient as symptoms of other diseases may be neglected or masked by the hydrocephalus-associated symptoms. Future research in brain ciliopathies may reveal new insights into brain disease in the broadest sense, given the essential role of cilia in neurodevelopment.

## Supplementary Material

fcad004_Supplementary_Data

## Data Availability

The data that support the findings of this study are available from the corresponding author, upon reasonable request.

## Abbreviations

α-DG =: alpha-dystroglycan
AF =: allele frequency
CPR =: Central Person Register
ICD =: International Classification of Diseases
iPSYCH =: Lundbeck Foundation Initiative for Integrative Psychiatric Research
MAF =: minor allele frequency
PCP =: planar cell polarity
PTV =: protein truncating variant
